# Supplementary material for: Genome-wide analysis of consistently RNA edited sites in human blood reveals interactions with mRNA processing genes and suggests correlations with cell types and biological variables
Source: BMC Genomics. 2018 Dec 27;19:963. doi: 10.1186/s12864-018-5364-8 (PMC6307200; doi:10.1186/s12864-018-5364-8)

Table S1. Summary statistics for the 33 highly edited sites with mean editing level above 0.9

Site id	Gene	Strand	Region	ALU	Minimum	Mean	Maximum
chr18_24179309	*KCTD1*	-	intronic	no	0.90	1.00	1.00
chr6_30453716	*RANP1*	+	intronic	no	0.94	1.00	1.00
chr18_24179333	*KCTD1*	-	intronic	no	0.91	1.00	1.00
chr18_24179324	*KCTD1*	-	intronic	no	0.91	1.00	1.00
chr9_90140405	*DAPK1*	+	intronic	no	0.92	1.00	1.00
chr6_132140917	*ENPP1*	+	intronic	no	0.90	1.00	1.00
chr15_25154978	*SNRPN*	+	intronic	no	0.30	1.00	1.00
chr16_27278365	*NSMCE1*	-	intronic	no	0.90	1.00	1.00
chr3_44619818	*RP11-944L7.4*	-	intronic	no	0.90	1.00	1.00
chr3_183868236	*EIF2B5*	+	intronic	no	0.93	0.99	1.00
chr20_3276419	*C20orf194*	-	ncRNA	no	0.92	0.99	1.00
chr5_130994333	*FNIP1*	-	intronic	no	0.82	0.99	1.00
chr7_38351411	*TARP*	-	intronic	no	0.90	0.99	1.00
chr3_44620372	*RP11-944L7.4*	-	intronic	no	0.82	0.99	1.00
chr22_42777186	*NFAM1*	-	3UTR	no	0.87	0.98	1.00
chr5_130994309	*FNIP1*	-	intronic	no	0.80	0.98	1.00
chr3_44620354	*RP11-944L7.4*	-	intronic	no	0.82	0.98	1.00
chr7_38351422	*TARP*	-	intronic	no	0.80	0.98	1.00
chr10_101992308	*CWF19L1*	-	3UTR	yes	0.80	0.98	1.00
chrX_70640516	*BCYRN1*	-	intronic	no	0.75	0.97	1.00
chr12_98943033	*TMPO*	+	3UTR	yes	0.76	0.96	1.00
chr5_130994344	*FNIP1*	-	intronic	no	0.77	0.96	1.00
chr19_56150729	*ZNF581*	+	intronic	yes	0.69	0.96	1.00
chr19_8557875	*PRAM1*	-	intronic	yes	0.80	0.95	1.00
chr1_160966434	*F11R*	-	3UTR	yes	0.79	0.95	1.00
chr6_31640793	*CSNK2B*	+	3UTR	yes	0.69	0.95	1.00
chr15_45025366	*TRIM69*	+	intronic	yes	0.7	0.95	1.00
chr7_128293538	*LINC01000*	+	ncRNA	no	0.77	0.94	1.00
chr14_20834467	*TEP1*	-	3UTR	no	0.67	0.93	1.00

chr22_42777158	*NFAM1*	-	3UTR	no	0.69	0.92	1.00
chr22_39350614	*APOBEC3A*	+	intronic	no	0.68	0.92	1.00
chr19_15772035	*CYP4F3*	+	3UTR	yes	0.66	0.91	1.00
chr15_90375494	*C15orf38*<br>*AP3S2*	-	3UTR	yes	0.70	0.90	1.00

Table S2. Cell composition variables associated to CES total editing rate and *ADAR* / *ADARB1* expression level.
The table reports number of subjects with non-zero value and results of association between cell composition variables and CES total editing rate or *ADAR* / *ADARB1* expression level (TPM). Pearson's product-moment correlation test were used to assess association. Pearson r coefficient is also reported. Variables measurable in less than 20 individuals (5% of total sample) were not analysed. Th = T helper, Tc = T cytotoxic, Tc_act = activated T cells, B = B linfocytes, NK = natural killer linfocytes, NK_act = activated natural killer linfocytes, mono = monocytes, DC = dendritic cells, DC_act = activated dendritic cells, neutro = neutrophils.

Cell variable	Tested variable	N. of subject with non zero value	P val	r
neutro	Total editing rate	452	1.78E-16	0.374
neutro	ADAR	452	7.31E-11	0.300
neutro	ADARB1	452	7.24E-19	-0.401
mono	Total editing rate	450	9.52E-07	0.229
mono	ADAR	450	5.38E-02	0.091
mono	ADARB1	450	7.30E-05	-0.186
Th	Total editing rate	452	3.46E-05	0.193
Th	ADAR	452	2.14E-02	-0.108
Th	ADARB1	452	3.69E-18	0.393
DC	Total editing rate	104	1.56E-04	0.362
DC	ADAR	104	6.16E-02	0.184
DC	ADARB1	104	3.08E-01	-0.101
NK	Total editing rate	407	2.87E-01	0.053
NK	ADAR	407	6.03E-03	0.136
NK	ADARB1	407	7.96E-02	0.087
Tc	Total editing rate	50	2.91E-01	0.152
Tc	ADAR	50	6.91E-01	-0.058
Tc	ADARB1	50	6.54E-01	-0.065
B	Total editing rate	389	7.79E-01	0.0143
B	ADAR	389	3.74E-02	-0.106
B	ADARB1	389	3.80E-21	0.454
DC_act	Total editing rate	11		
DC_act	ADAR	11		
DC_act	ADARB1	11		
NK_act	Total editing rate	8		
NK_act	ADAR	8		
NK_act	ADARB1	8		
Tc_act	Total editing rate	1		
Tc_act	ADAR	1		
Tc_act	ADARB1	1		

Table S3. Phenotypic / pharmacological variables considered in the study.
The table reports the 28 variables included in the study, extracted from the original dataset described in [29].

Variable	Explanation	Included as covariate
Age	Age at recruitment	
BMI current	BMI at the time of recruitment (self-report)	x
BMI max	Maximum BMI lifetime (self-report)	
Region	Region of country	
Sex	Sex	x
Ate before	Ate before blood draw (self-report)	
Exercise before	Exercised before blood draw (self-report)	
Smoke before	Smoked before blood draw (self-report)	
Time of draw	Time blood was drawn converted to numeric eg, 2:30 pm = 14.5	
All drugs	Used any drug (cannabis, cocaine, hallucinogens, stimulants)	
Cannabis	Used cannabis	
Cocaine	Used cocaine	
Hallucinogens	Used hallucinogens	
Stimulants	Used stimulants	
ACE inhibitor	Currently use ACE inhibitors	x
Blood pressure meds	Currently use blood pressure medications	x
Oral birth-control pill	Currently use oral birth control pill	x
Cholesterol lowering	Currently use cholesterol lowering medications	
Anti histamine	Currently use anti histamine medications	
Thyroid meds	Currently use thyroid hormone supplementation	x
Proton-pump inhibitor	Currently use proton pump inhibitors	
None treatments	None medical treatments	
Alcohol abuse	Ever had alcohol abuse	
Current alcohol use	Level of current alcohol use	
100 cigs lifetime	Ever smoked 100 cigarettes lifetime	
Currently smoke	Currently smoke	
N daily cigarettes	Current typical number of cigarettes smoked daily	
Fager life total	Total Fagerstrom score, worst lifetime period	

Table S4. Biological / pharmacological factors associated to CES total editing rate and ADARs expression

The table reports association results between biological / pharmacological factors and CES total editing rate or *ADAR* / *ADARB1* expression level (calculated as Transcript per Million). Kruskal-Wallis test, Mann-Whitney-Wilcoxon test and Pearson's product-moment correlation test were used to assess association for categorical, binary and continuous variables, respectively. For continuous variables Pearson r coefficient is also reported. We also calculated a cell adjusted P value, that represents significance of LRT test for impact of each variable after accounting for cell factors associated to CES total editing rate (neutrophils, monocytes, DC, Th). Variables are sorted based on P value of association with total editing rate.

Variable	Tested association	P value	Cell adjusted P value	r
Blood pressure meds	Total editing rate	3.95E-04	8.93E-04	
Blood pressure meds	ADAR	5.02E-04	1.97E-02	
Blood pressure meds	ADARB1	9.62E-01	4.52E-01	
BMI current	Total editing rate	1.68E-03	4.77E-02	-0.148
BMI current	ADAR	3.68E-03	2.29E-02	-0.137
BMI current	ADARB1	2.95E-01	9.31E-02	-0.050
Age	Total editing rate	3.62E-03	7.57E-03	-0.137
Age	ADAR	1.47E-03	5.29E-03	-0.149
Age	ADARB1	2.99E-01	1.17E-01	-0.049
BMI max	Total editing rate	4.64E-03	7.44E-02	-0.134
BMI max	ADAR	6.49E-03	3.18E-02	-0.128
BMI max	ADARB1	3.32E-01	1.24E-01	-0.046
Sex	Total editing rate	4.12E-02	3.53E-01	
Sex	ADAR	2.23E-01	3.93E-02	
Sex	ADARB1	1.72E-01	2.52E-02	
Oral birth-control pill	Total editing rate	5.98E-02	1.54E-01	
Oral birth-control pill	ADAR	1.75E-01	5.85E-01	
Oral birth-control pill	ADARB1	5.95E-01	3.47E-01	
Alcohol abuse	Total editing rate	6.96E-02	4.21E-01	
Alcohol abuse	ADAR	1.58E-01	2.97E-01	
Alcohol abuse	ADARB1	9.82E-01	4.42E-01	
Cocaine	Total editing rate	1.54E-01	9.01E-01	
Cocaine	ADAR	7.78E-01	3.81E-01	
Cocaine	ADARB1	5.46E-01	6.87E-01	
ACE inhibitor	Total editing rate	2.37E-01	6.04E-01	
ACE inhibitor	ADAR	3.93E-01	8.91E-01	
ACE inhibitor	ADARB1	4.29E-01	9.57E-01	
Region	Total editing rate	2.46E-01	1.09E-01	
Region	ADAR	7.79E-01	7.07E-01	

Region	ADARB1	6.10E-01	7.24E-01	
Hallucinogens	Total editing rate	2.46E-01	7.30E-01	
Hallucinogens	ADAR	2.51E-01	5.18E-02	
Hallucinogens	ADARB1	5.94E-02	2.29E-01	
Cholesterol lowering	Total editing rate	2.51E-01	5.59E-01	
Cholesterol lowering	ADAR	6.33E-01	8.00E-01	
Cholesterol lowering	ADARB1	8.66E-01	9.84E-01	
None treatments	Total editing rate	3.20E-01	4.42E-01	
None treatments	ADAR	8.89E-01	7.14E-01	
None treatments	ADARB1	2.39E-01	2.87E-01	
100 cigs lifetime	Total editing rate	3.63E-01	7.39E-01	
100 cigs lifetime	ADAR	3.68E-01	9.71E-01	
100 cigs lifetime	ADARB1	9.65E-01	9.12E-02	
All drugs	Total editing rate	3.91E-01	4.61E-01	
All drugs	ADAR	4.69E-01	2.65E-01	
All drugs	ADARB1	3.49E-01	3.49E-01	
Cannabis	Total editing rate	4.53E-01	5.51E-01	
Cannabis	ADAR	6.76E-01	4.31E-01	
Cannabis	ADARB1	1.64E-01	1.81E-01	
Current alcohol use	Total editing rate	8.32E-02	8.03E-02	
Current alcohol use	ADAR	4.44E-01	1.17E-01	
Current alcohol use	ADARB1	3.62E-01	5.83E-01	
Stimulants	Total editing rate	5.42E-01	9.65E-01	
Stimulants	ADAR	8.95E-01	3.71E-01	
Stimulants	ADARB1	1.64E-01	4.86E-01	
Time of draw	Total editing rate	6.02E-01	4.66E-01	-0.025
Time of draw	ADAR	6.12E-01	8.23E-01	-0.024
Time of draw	ADARB1	9.37E-03	4.82E-02	0.122
Thyroid meds	Total editing rate	6.08E-01	7.65E-01	
Thyroid meds	ADAR	4.98E-01	3.49E-01	
Thyroid meds	ADARB1	1.12E-02	4.17E-02	
N daily cigarettes	Total editing rate	6.17E-01	4.12E-01	0.024
N daily cigarettes	ADAR	9.46E-01	2.70E-01	-0.003
N daily cigarettes	ADARB1	1.49E-01	9.40E-01	0.068
Fager life total	Total editing rate	6.26E-01	4.91E-01	-0.023
Fager life total	ADAR	7.35E-02	2.49E-01	-0.084
Fager life total	ADARB1	5.62E-01	5.70E-02	-0.027
Ate before	Total editing rate	6.71E-01	5.66E-01	
Ate before	ADAR	3.23E-01	3.60E-01	

Ate before	ADARB1	4.92E-02	1.42E-02	
Smoke before	Total editing rate	7.02E-01	3.59E-01	
Smoke before	ADAR	8.21E-01	5.42E-01	
Smoke before	ADARB1	8.97E-02	6.96E-01	
Currently smoke	Total editing rate	7.34E-01	4.13E-01	
Currently smoke	ADAR	8.97E-01	5.84E-02	
Currently smoke	ADARB1	9.26E-02	8.89E-01	
Anti histamine	Total editing rate	7.44E-01	2.75E-01	
Anti histamine	ADAR	6.67E-01	7.67E-01	
Anti histamine	ADARB1	8.09E-01	9.46E-01	
Exercise before	Total editing rate	9.50E-01	2.53E-01	
Exercise before	ADAR	1.00E+00	2.59E-01	
Exercise before	ADARB1	8.94E-01	8.78E-01	
Proton-pump inhibitor	Total editing rate	9.60E-01	1.08E-01	
Proton-pump inhibitor	ADAR	6.29E-01	1.44E-01	
Proton-pump inhibitor	ADARB1	1.77E-01	3.44E-02	

Table S5. Association of cell composition variables, biological and pharmacological variables with the top 5 editing principal components.
For cell types variables, the table reports p-value for association with the first 5 PCs, calculated using Pearson's product-moment correlation test. For biological / pharmacological variables we reported the p-value for association after correcting for the 4 cell variables associated to CES total editing rate (neutrophils, monocytes, DC, Th; see Table S2). Variable are sorted based on association with PC1.

**Cell composition variables**					
**variable**	**PC1**	**PC2**	**PC3**	**PC4**	**PC5**
neutrophils	7.82E-44	7.50E-02	2.02E-08	3.06E-16	1.93E-09
monocytes	1.71E-08	9.55E-03	1.45E-09	1.92E-01	6.24E-09
B	9.70E-05	2.82E-08	9.61E-01	3.04E-08	1.77E-01
DC	1.91E-03	6.59E-02	1.87E-03	1.37E-04	1.62E-01
Tc	3.60E-01	7.66E-01	2.37E-02	9.86E-03	5.33E-01
Th	2.51E-01	3.31E-20	1.14E-01	1.13E-06	5.45E-01
NK	7.83E-01	3.55E-06	3.89E-03	1.81E-01	4.04E-04
**Biological / pharmacological variables after correction for cell composition**					
**variable**	**PC1**	**PC2**	**PC3**	**PC4**	**PC5**
Blood pressure meds	7.45E-03	9.56E-01	4.27E-01	9.58E-01	5.22E-01
Current alcohol use	9.31E-03	9.40E-01	6.60E-01	1.40E-01	2.92E-01
Age	1.70E-02	7.65E-01	2.15E-01	2.91E-01	3.38E-01
Time of draw	4.24E-02	9.41E-03	8.84E-02	5.11E-01	1.94E-02
Exercise before	4.29E-02	8.35E-01	1.75E-01	3.69E-01	6.43E-01
BMI current	4.73E-02	8.91E-01	2.35E-01	5.84E-02	1.21E-01
N daily cigarettes	4.77E-02	7.17E-01	9.74E-01	1.88E-01	5.87E-01
BMI max	7.26E-02	8.71E-01	3.88E-01	7.99E-02	1.63E-01
Currently smoke	7.65E-02	8.45E-01	6.60E-01	1.82E-01	2.30E-01
Sex	1.90E-01	1.01E-02	1.72E-02	2.13E-02	5.39E-11
Smoke before	1.94E-01	4.18E-01	7.86E-01	5.89E-01	3.21E-01
Alcohol abuse	2.31E-01	3.57E-01	1.94E-01	3.80E-01	6.49E-01
Proton-pump inhibitor	2.40E-01	5.29E-01	2.94E-01	9.63E-01	7.58E-01
All drugs	2.82E-01	9.84E-01	3.55E-01	7.33E-02	4.26E-01
Region	3.07E-01	7.86E-01	9.49E-01	1.34E-01	6.46E-01
Oral birth-control pill	3.90E-01	4.49E-01	3.80E-01	1.75E-01	7.55E-01
Cannabis	4.53E-01	8.98E-01	4.39E-01	1.97E-01	4.84E-01
100 cigs lifetime	4.55E-01	2.12E-01	7.28E-01	3.55E-01	4.33E-01
Ate before	5.85E-01	3.52E-02	3.19E-01	9.69E-01	3.16E-01
Anti histamine	6.24E-01	4.96E-01	8.12E-01	8.80E-01	9.66E-01
ACE inhibitor	6.50E-01	3.93E-01	5.79E-01	5.64E-01	1.91E-01
Stimulants	6.97E-01	7.34E-01	5.63E-01	8.30E-01	2.69E-01

Thyroid meds	7.64E-01	1.42E-01	3.82E-01	8.34E-01	1.80E-02
None treatments	8.39E-01	4.25E-01	8.03E-01	8.55E-01	6.62E-01
Cholesterol lowering	9.11E-01	4.22E-01	5.33E-01	5.42E-02	2.42E-02
Cocaine	9.15E-01	6.00E-01	4.84E-01	4.46E-01	4.79E-01
Hallucinogens	9.94E-01	1.78E-01	1.43E-01	3.85E-01	4.62E-01

Table S6. Association of drug an medication intake variables with sex of subjects. Association was analyzed by Chi-Square test.

Variable	P-value
Oral birth-control pill meds	3.00E-06
Alcohol abuse	4.00E-05
Cholesterol lowering	0.001
Thyroid meds	0.005
Current alcohol use	0.02
All drugs	0.2
Hallucinogens	0.2
None treatments	0.2
100 cigs lifetime	0.2
Blood pressure meds	0.3
N daily cigarettes	0.3
Stimulants	0.3
Cocaine	0.4
ACE Inhibitor	0.5
Cannabis	0.5
Smoke before	0.6
Proton-pump inhibitor	0.7
Currently smoke	0.8
Anti histamine	0.9

Table S7. Association of ADAR and ADARB1 expression level with the top 5 editing principal components.
The table reports p-value for association of ADAR and ADARB1 expression level with the first 5 PCs, calculated using Pearson's product-moment correlation test.

**Gene**	**PC1**	**PC2**	**PC3**	**PC4**	**PC5**
ADAR	3.07E-58	4.66E-02	4.09E-07	1.61E-09	3.17E-03
ADARB1	1.41E-01	2.10E-42	1.86E-03	1.13E-01	1.32E-05

Table S8. Results of association with CES total editing rate for the known ADAR eQTLs. The rank among the 734,251 tested SNPs is reported.

Rank	SNP	A1	BETA	P-value
58	rs903323	A	-2.47E-03	4.19E-05
59	rs6699825	G	-2.47E-03	4.19E-05
64	rs9426830	G	-2.45E-03	4.76E-05
124	rs1127313	A	-2.30E-03	1.22E-04
154	rs1127311	A	-2.28E-03	1.44E-04
480	rs9427108	A	-2.09E-03	5.58E-04
658	rs2335230	C	2.61E-03	8.04E-04
1032	rs9427114	G	-1.93E-03	1.33E-03
3052	rs9427097	C	-2.24E-03	4.03E-03
4316	rs11264248	G	1.66E-03	5.74E-03
4319	rs7547072	A	-1.66E-03	5.75E-03
15375	rs1876304	A	1.68E-03	2.09E-02
63347	rs2072660	A	1.23E-03	8.67E-02
87416	rs7556080	A	1.07E-03	1.20E-01
136823	rs2131902	G	8.91E-04	1.87E-01
168738	rs1127309	A	8.18E-04	2.30E-01
170542	rs9426823	A	8.09E-04	2.33E-01
172939	rs2229857	A	8.05E-04	2.36E-01
174891	rs6656743	T	7.98E-04	2.39E-01
174972	rs7534678	A	7.96E-04	2.39E-01
210425	rs11264235	A	7.22E-04	2.87E-01
211471	rs7554577	A	6.71E-04	2.89E-01
219063	rs1127314	G	7.02E-04	2.99E-01
241948	rs11264222	A	6.64E-04	3.30E-01
295150	rs884617	A	5.47E-04	4.03E-01
319610	rs6696760	A	6.89E-04	4.36E-01
326377	rs2172706	G	-5.56E-04	4.45E-01
414576	rs4845617	A	-3.63E-04	5.65E-01
420648	rs10908431	G	3.71E-04	5.73E-01
506659	rs2297607	G	-2.95E-04	6.90E-01
530232	rs1194587	G	2.14E-04	7.22E-01
531916	rs952146	G	-2.27E-04	7.25E-01
534952	rs3738032	A	-2.57E-04	7.29E-01
575493	rs2988721	A	-1.92E-04	7.84E-01
581493	rs10908835	G	-1.70E-04	7.92E-01
668056	rs2274988	A	-7.97E-05	9.10E-01

Figure S1. Measurable editing sites in our dataset cover most sites in blood expressed genes
We calculated the fraction of total editing sites reported from RADAR that have adequate coverage (at least 10X) in our dataset. Based on expression data from GTeX v7 we calculated this fraction stratified based on most expressed genes in whole blood. Our dataset covers > 75% of total RADAR sites in the top 5,000 genes expressed in whole blood, supporting our ability to investigate editing events in this tissue.

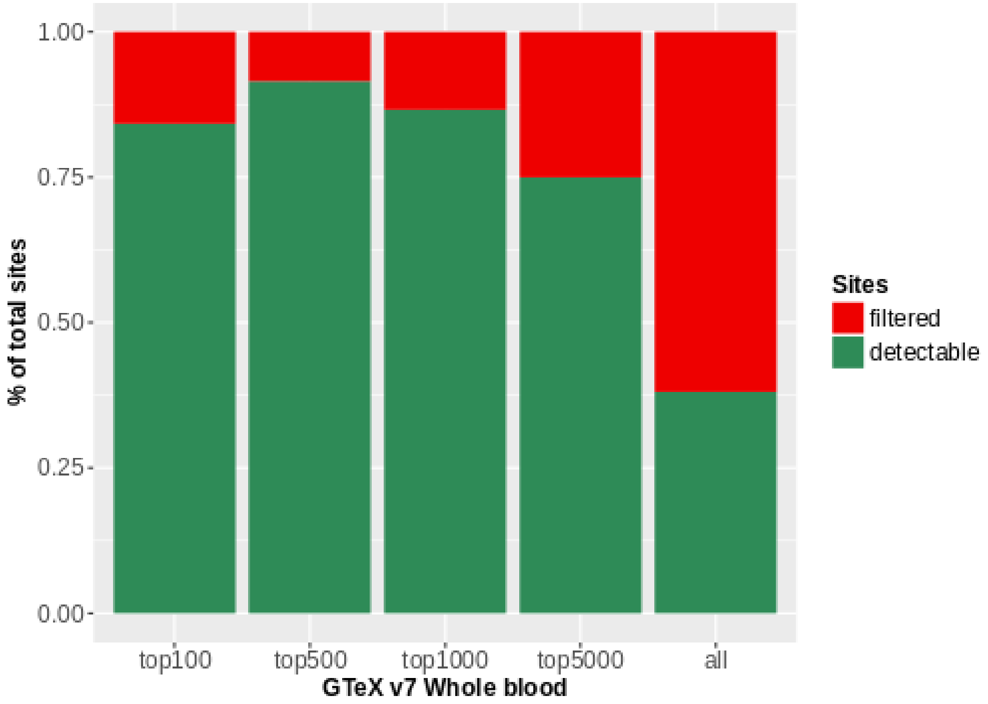

Figure S2. Distribution of sites with detectable editing

Considering the 709,184 sites addressable in our dataset, we calculated for each one the number of individuals with detectable editing level. The distribution of number of sites detectable in at least N samples is represented in the plot. We found that 691,304 sites have no detectable editing and most sites have a detectable editing level only in a small fraction of samples. The red dot indicates the number of sites with detectable editing level in at least 100 individuals.

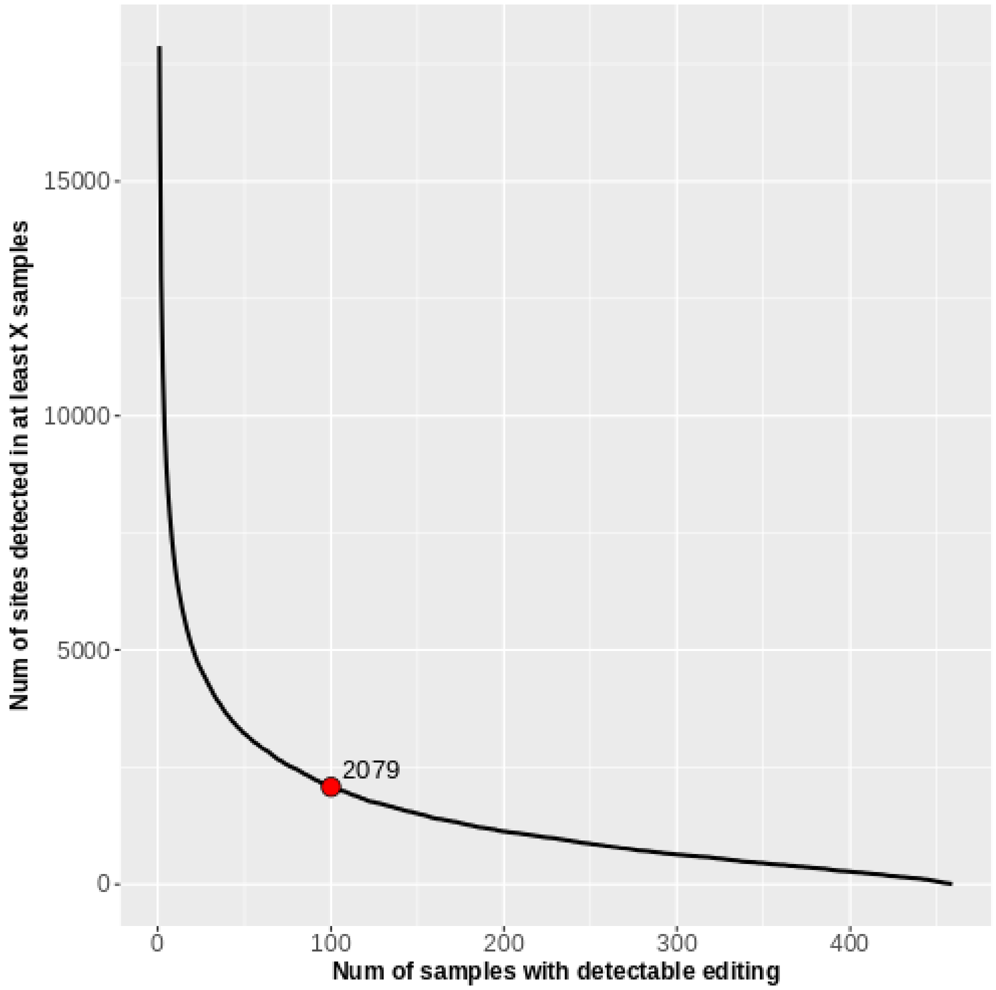

Figure S3. Concordance between editing levels observed in our study and REDIPortal
We calculated concordance correlation coefficient (CCC) between mean editing values for the 2,003 overlapping sites detected in our data (Mean) and also reported by REDIPortal (Mean REDI). The blue dashed line represents the fitted linear model, while red line represents linear model for perfect concordance.

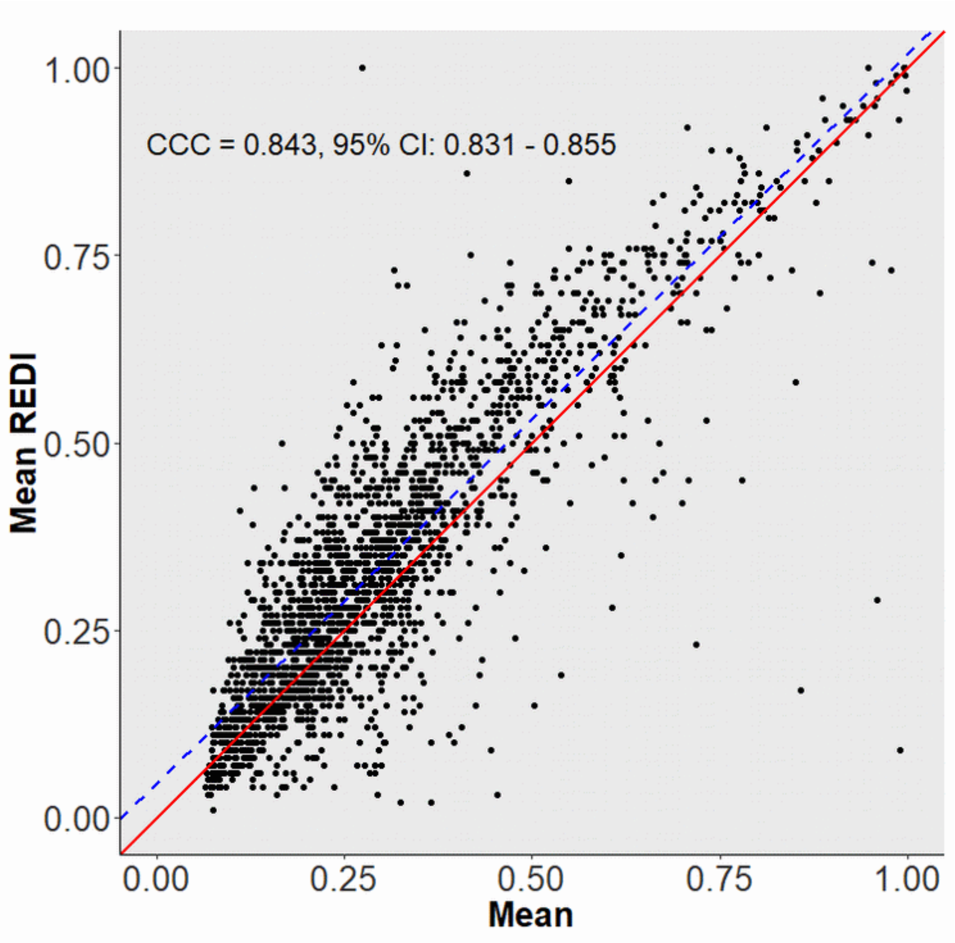

Figure S4. Overlap between CES and known miRNA binding sites from TargetScan
We found a total of 495 CES located within known miRNA binding sites from TargetScan v.7.2. The analysis was performed separately for broadly conserved, conserved and non conserved miRNA and miRNA binding site groups, as defined by TargetScan.

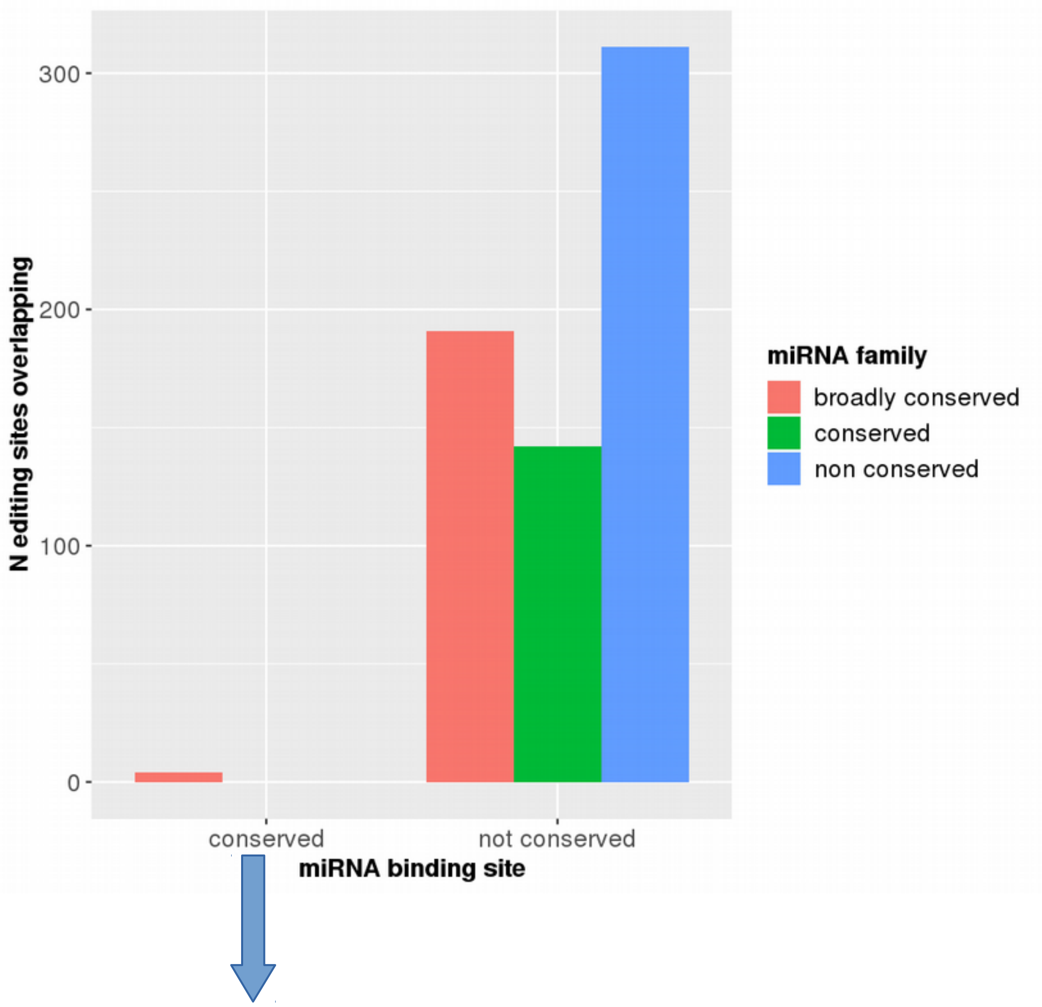

chr6	42905086	CNPY3
chr6	42905085	CNPY3
chr6	42905211	CNPY3
chr1	184762472	FAM129A

Figure S5. Correlation of editing levels between sites

Using Spearman rank test, we analyzed the correlation between editing levels among the 2,079 trusted sites. Significant correlations (FDR < 0.05) was generally low (a,d), except for close sites within 50 bp (b), for which we observed strong positive correlations (rho > 0.5). An example of editing island within 50bp on chromosome 3 is reported in correlation plot (c). Circle dimensions and color scale represent rho values of site-site correlation. Correlation and p-values for all relationship fir FDR < 0.05 are plotted in (d), with point colored according to distance between the sites considered. For the 66 couples of sites showing high correlations (rho > 0.5), the distributions of absolute difference in editing levels across subjects are reported in (e).

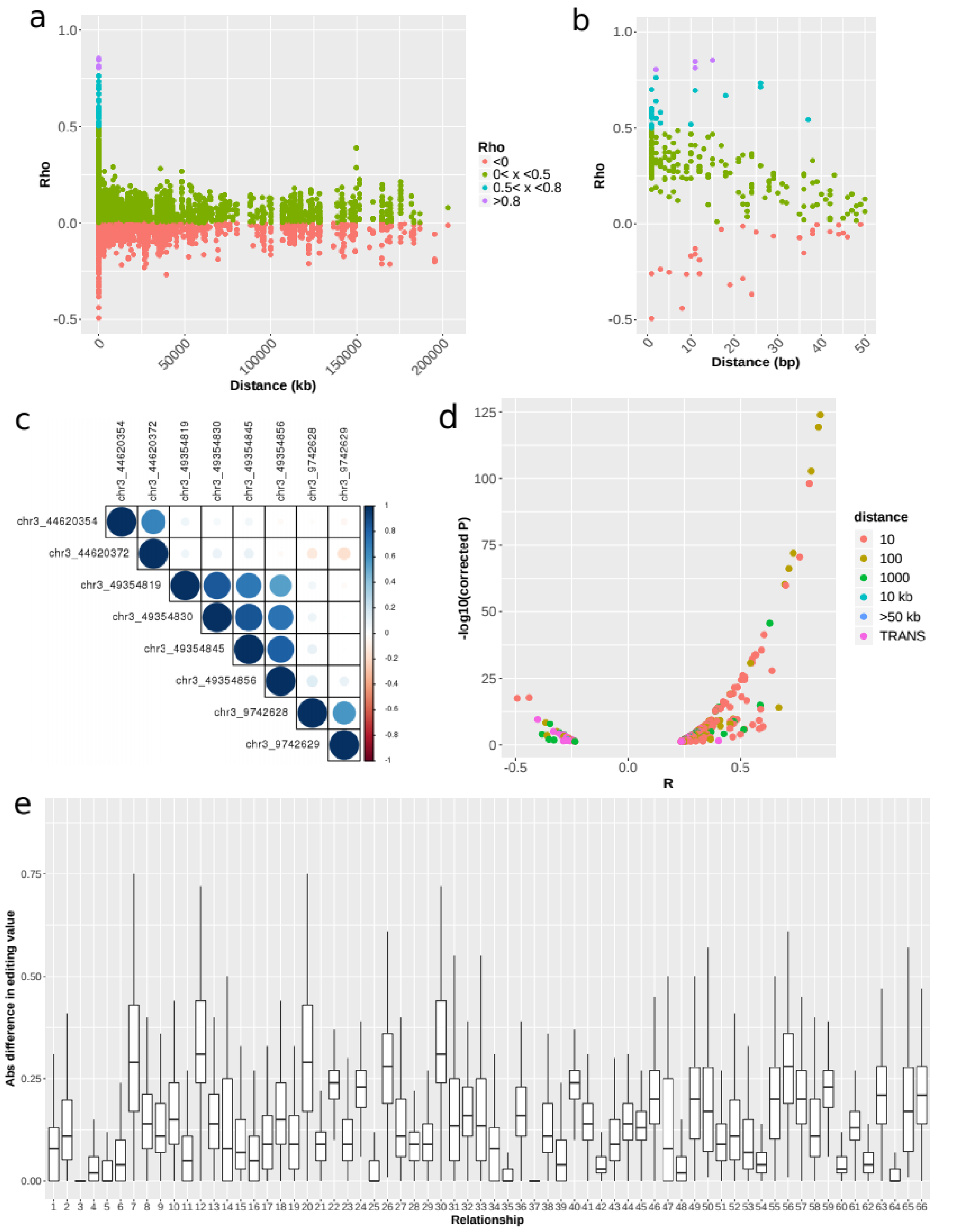

Figure S6. Correlation of *ADAR* and *ADARB1* expression on Alu and Non-Alu editing sites
We used robust regression analysis to estimate the association between *ADAR* (a) or *ADARB1* (b) expression and CES total editing rate, considering Alu and non-Alu sites separately. The graphs report adjusted p-value and R^2 value from robust regression analysis. *ADAR* showed association with both class of sites, while *ADARB1* showed no significant associations.

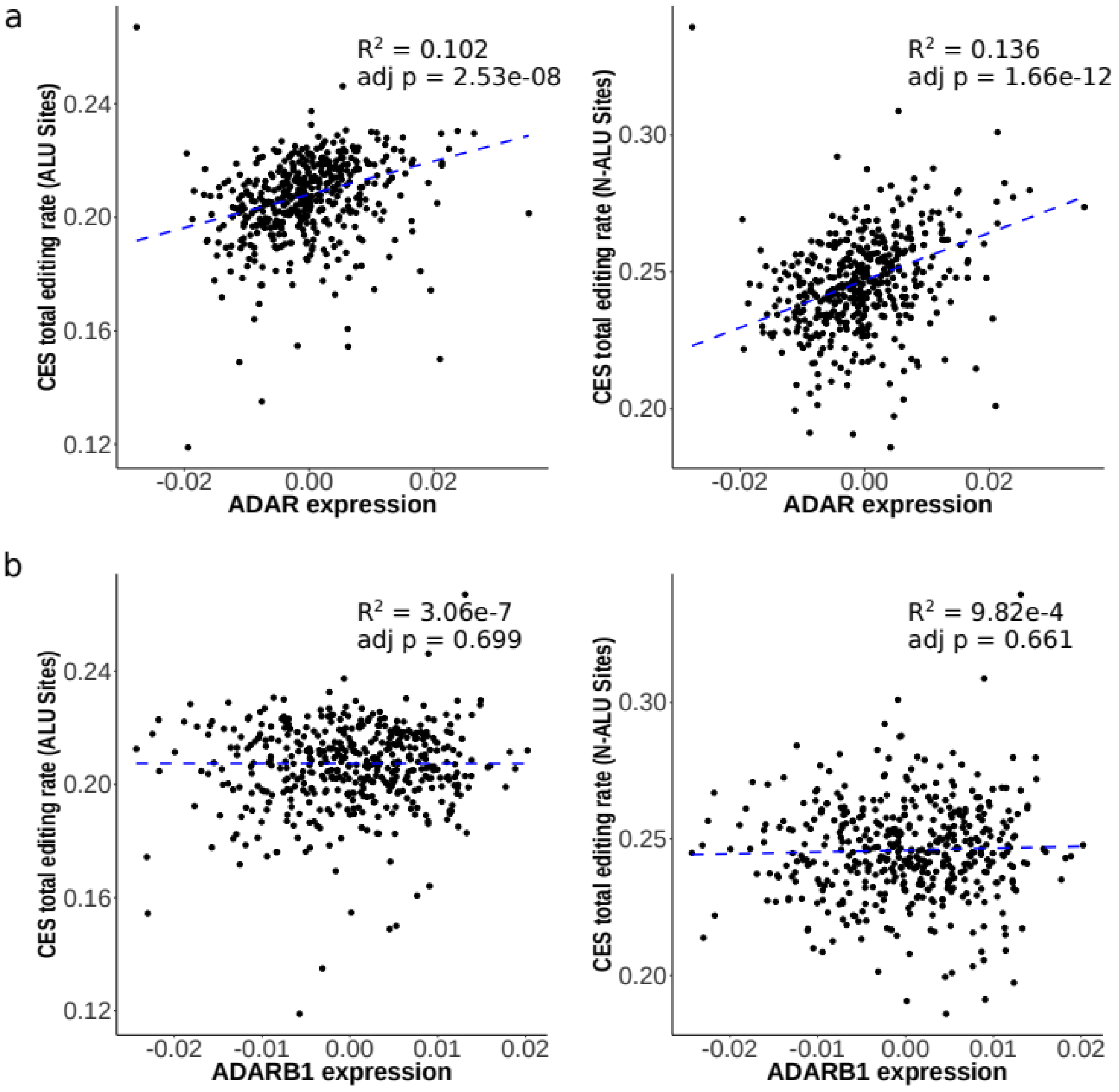

Figure S7. Linear regression models predicting *ADAR* / *ADARB1* expression level from cell composition variables and biological variables

To better evaluate the impact of cellular composition on *ADAR* / *ADARB1* expression, we computed a liner regression model predicting gene expression from the 7 cell variables (in red). We observed high correlation between predicted and expected values especially fro *ADARB1*. Adding the biological variables associated to *ADAR* / *ADARB1* expression (see Table S4) improve the prediction model for *ADAR*, but not *ADARB1* (in green).

R-squared values for each model are reported with the corresponding color. P-value refers to the LRT test between the model with and without the biological variables.

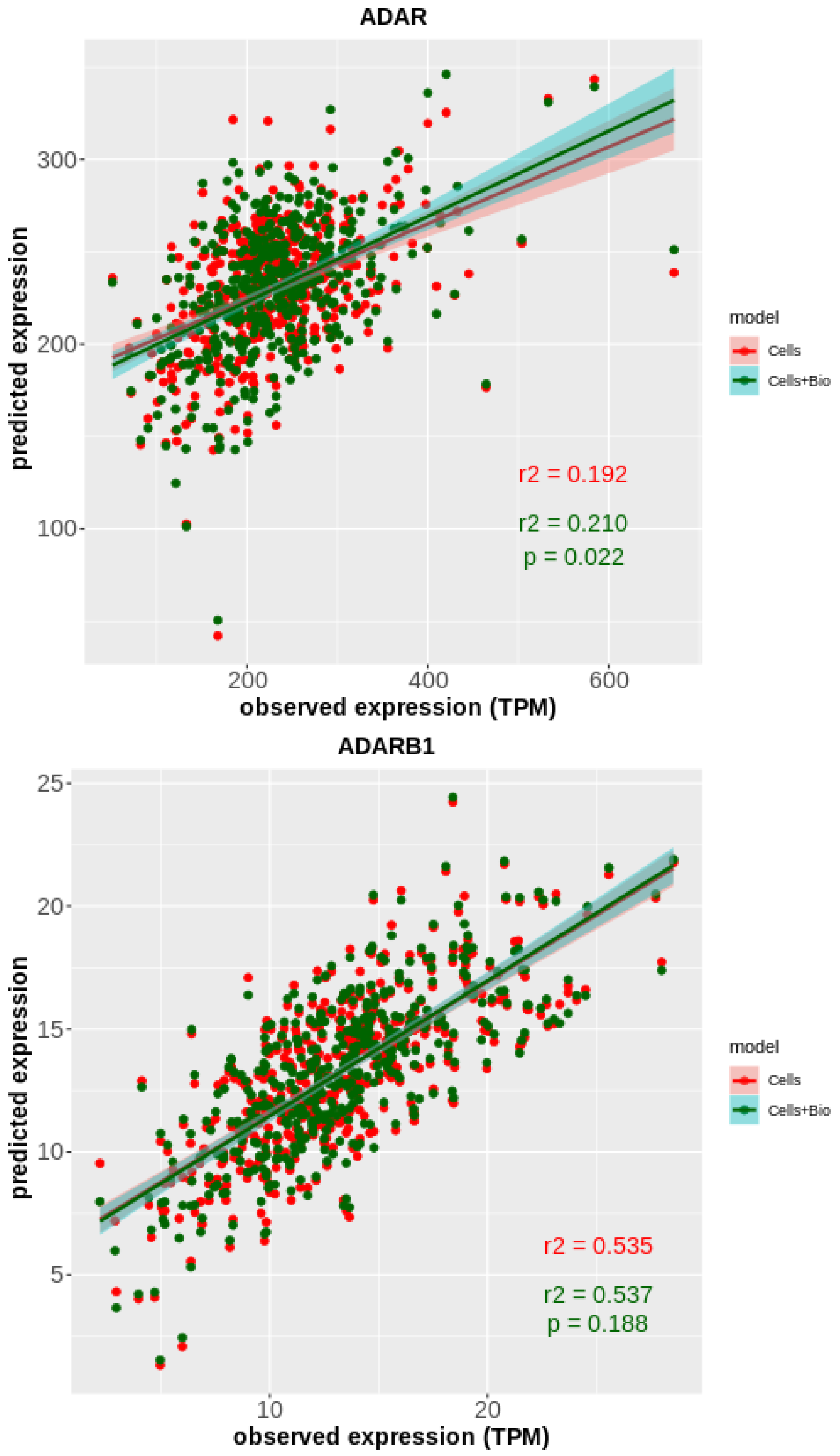

Figure S8. Effect of the top associated *ADAR* eQTL (rs6699825) on *ADAR* expression and CES total editing rate.

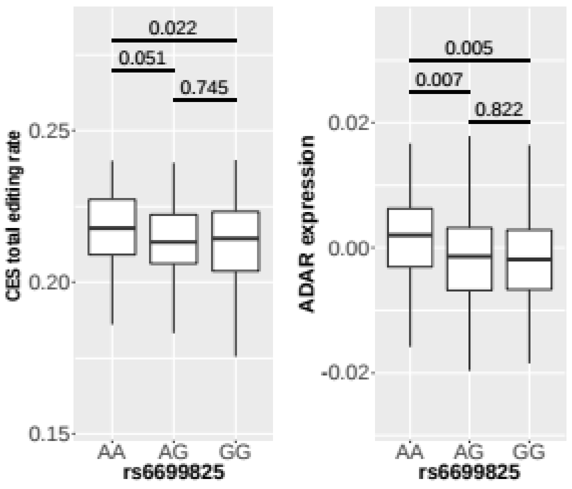

Figure S9. Distribution of the main RNA sequencing metrics for experiments used in this study.

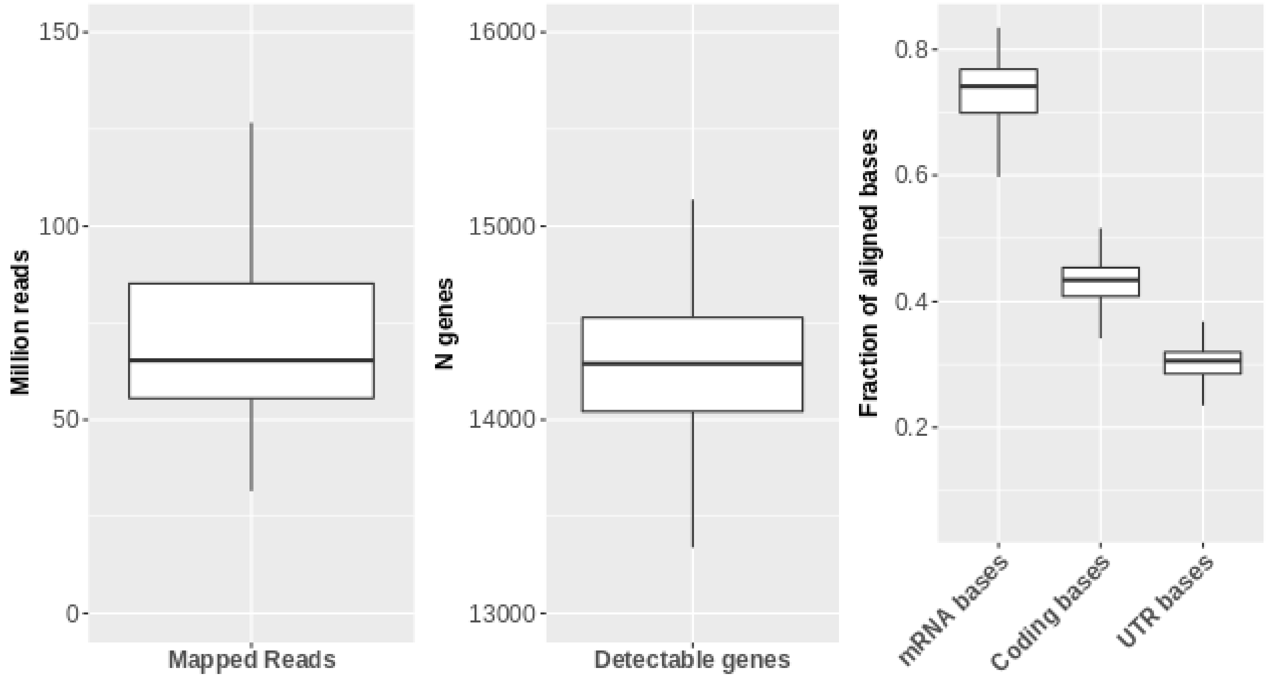

Figure S10. Correlation between observed editing variance and median coverage at editing sites. For single editing sites, we observed a strong correlation between median coverage and the observed variance, so that sites with lower coverage resulted in higher variance. The correlation seems to disappear for sites above 40 X coverage (blue dots). This suggest that site coverage should be taken into account when performing associations on editing levels.

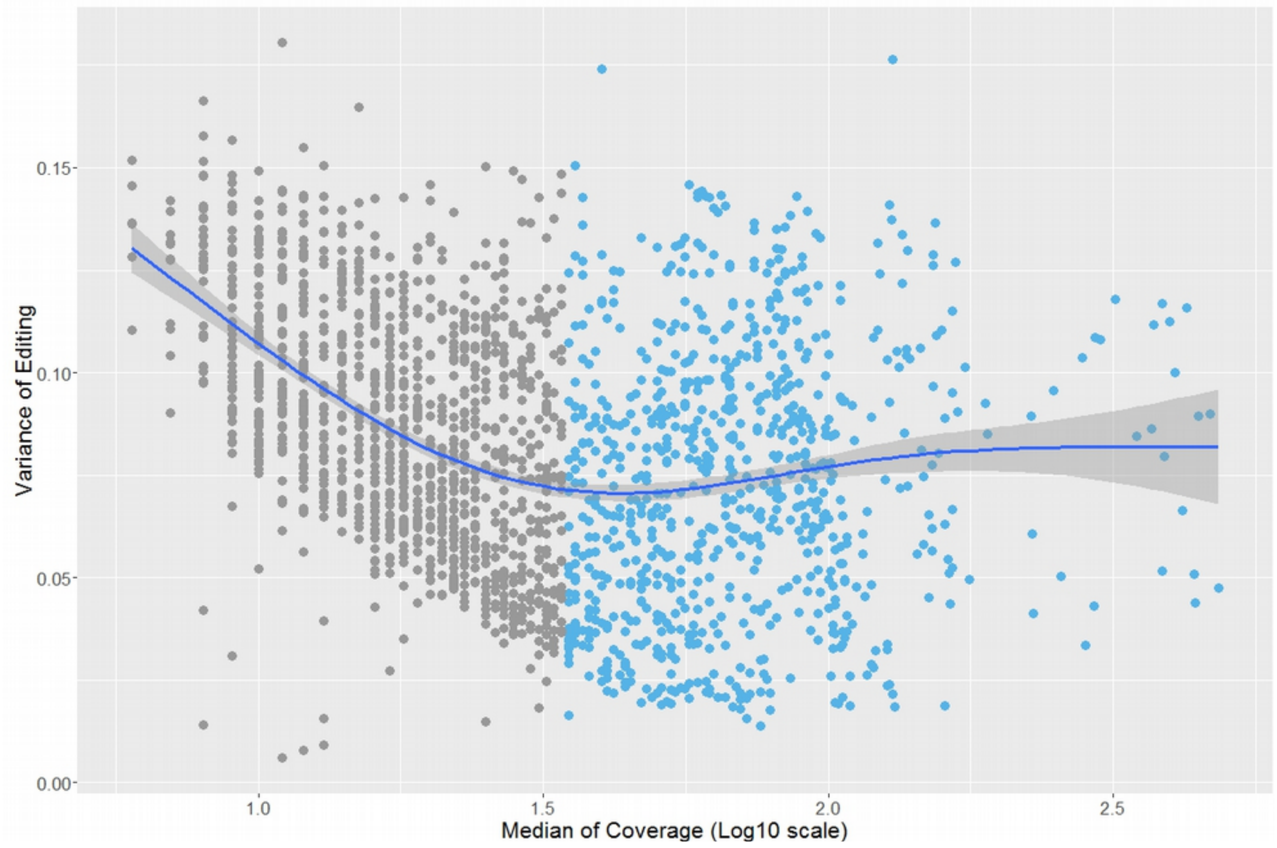

Supplement: Supplementary file 1 — Table S1. Summary statistics for the 33 highly edited sites. Table S2. Cell composition variables associated to CES total editing rate and ADAR / ADARB1 expression level. Table S3. Phenotypic / pharmacological variables considered in the study. Table S4. Biological / pharmacological factors associated to CES total editing rate and ADARs expression. Table S5. Association of cell composition variables, biological and pharmacological variables with. The top 5 editing principal components. Table S6. Association of drug an medication intake variables with sex of subjects. Table S7. Association of ADAR and ADARB1 expression with the top 5 editing principal components. Table S8. Association with CES total editing rate for the known ADAR eQTLs. Figure S1. Measurable editing sites cover most sites in blood expressed genes. Figure S2. Distribution of sites with detectable editing. Figure S3. Concordance between editing levels and REDIPortal. Figure S4. Overlap between CES and known miRNA binding sites from TargetScan. Figure S5. Correlation of editing levels between sites. Figure S6. Correlation of ADAR and ADARB1 expression on Alu and Non-Alu editing sites. Figure S7. Linear regression models predicting ADAR / ADARB1 expression level from cell composition variables and biological variables. Figure S8. Effect of the top associated ADAR eQTL on ADAR expression and CES total editing rate. Figure S9. Distribution of the main RNA sequencing metrics for the experiments used. Figure S10. Correlation between observed editing variance and median coverage at editing sites. (PDF 1157 kb) [file 12864_2018_5364_MOESM1_ESM.pdf]
